# Aptamer-Targeted Dendrimersomes Assembled from Azido-Modified Janus Dendrimers “Clicked” to DNA

**DOI:** 10.1021/acs.biomac.3c01108

**Published:** 2024-02-23

**Authors:** Paige Bristow, Kyle Schantz, Zoe Moosbrugger, Kailey Martin, Haley Liebenberg, Stefan Steimle, Qi Xiao, Virgil Percec, Samantha E. Wilner

**Affiliations:** † Department of Chemistry, Ursinus College, Collegeville, Pennsylvania 19426, United States; ‡ Department of Biochemistry and Biophysics, Perelman School of Medicine, University of Pennsylvania, Philadelphia, Pennsylvania 19014, United States; § Roy & Diana Vagelos Laboratories, Department of Chemistry, University of Pennsylvania, Philadelphia, Pennsylvania 19014, United States

## Abstract

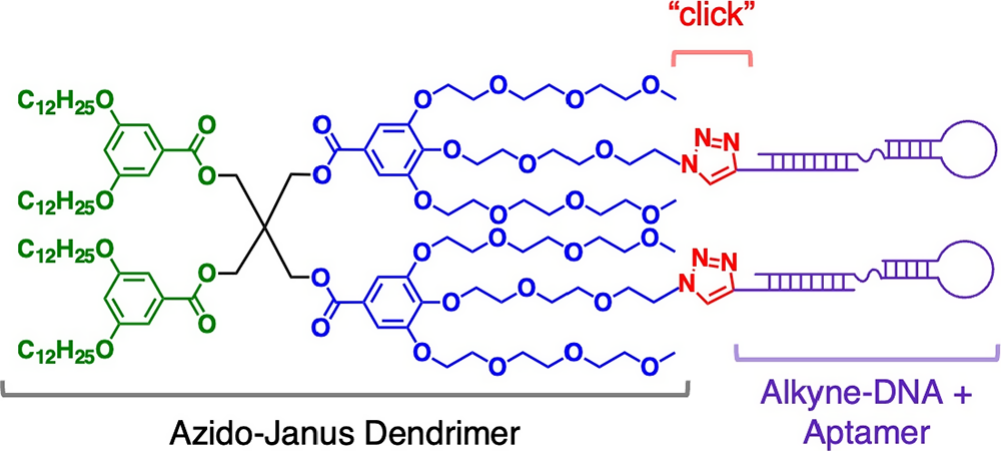

Amphiphilic Janus dendrimers (JDs), synthetic alternatives to lipids, have the potential to expand the scope of nanocarrier delivery systems. JDs self-assemble into vesicles called dendrimersomes, encapsulate both hydrophobic cargo and nucleic acids, and demonstrate enhanced stability in comparison to lipid nanoparticles (LNPs). Here, we report the ability to enhance the cellular uptake of Janus dendrimersomes using DNA aptamers. Azido-modified JDs were synthesized and conjugated to alkyne-modified DNAs using copper-catalyzed azide alkyne cycloaddition. DNA-functionalized JDs form nanometer-sized dendrimersomes in aqueous solution via thin film hydration. These vesicles, now displaying short DNAs, are then hybridized to transferrin receptor binding DNA aptamers. Aptamer-targeted dendrimersomes show improved cellular uptake as compared to control vesicles via fluorescence microscopy and flow cytometry. This work demonstrates the versatility of using click chemistry to conjugate functionalized JDs with biologically relevant molecules and the feasibility of targeting DNA-modified dendrimersomes for drug delivery applications.

## Introduction

Drug delivery technology addresses limitations associated with conventional administration of therapeutics. Nanocarriers, in particular, have been utilized to improve stability, solubility, distribution, intracellular transport, and efficacy of encapsulated drugs.^1^ Lipid nanoparticles (LNPs) are especially popular delivery vehicles due to their amphipathic nature, making it possible to encapsulate diverse cargoes ranging from hydrophobic small-molecule drugs such as doxorubicin^2^ to hydrophilic nucleic acids including mRNA (mRNA).^3^ LNPs serve to shield these cargoes from degradation and enhance their cellular uptake, which would otherwise be hindered due to poor solubility in physiological systems or inefficient cell membrane permeability. In fact, LNPs represent 52% of nanoparticles assessed in clinical trials between 2016 and 2021.^4^ Despite the success of some LNP formulations in the clinic, challenges with this technology remain, including inefficient intracellular delivery,^5^ undesirable storage conditions required to maintain LNP integrity,^6,7^ and ineffective transport to tissues other than the liver.^8^

Other delivery systems have the potential to address these challenges. Amphiphilic Janus dendrimers (JDs), which are constructed from two distinct hydrophobic and hydrophilic minidendrons, provide a synthetic alternative to lipids in the construction of nanocarriers.^9^ Due to their amphipathic structure, JDs self-assemble into bilayer structures called dendrimersomes.^9^ Dendrimersomes are uniform in size, exhibit mechanical stability as well as stability in serum, and can encapsulate a range of therapeutic cargoes.^9−15^ These cargoes include anticancer drugs such as doxorubicin^9,15,16^ as well as nucleic acid therapeutics including mRNA^10,12,17^ for vaccine development and DNA plasmids for gene delivery.^18−20^ Furthermore, JDs exhibit limited toxicity as demonstrated in vitro via cell viability assays^9,21,22^ and in vivo via assessment of organ function, blood biochemistry, and expression of inflammatory cytokines.^22^ Dendrimersome delivery systems investigated in vivo to date similarly demonstrate biocompatibility.^10,12,15,17,23^ When compared to stealth liposomes made of phospholipids, dendrimersomes exhibit superior encapsulation and retention of hydrophobic cargo, further suggesting their utility in drug delivery.^14^ Importantly, the modularity of JD synthesis allows for the incorporation of functional groups within the hydrophilic dendron.^13^ Such modifications provide the opportunity for the introduction of targeting ligands to the dendrimersome surface, with the potential to create a delivery vehicle that exhibits improved uptake in ligand-binding cells. To date, isothiocyanate-functionalized JDs have been conjugated to amine-modified glycans as a platform to mimic the cell membrane glycocalyx; however, covalent attachment of other biomolecules to JDs is yet to be explored.^13^

Nucleic acids are a natural choice for bioconjugation owing to their biocompatibility, ease of chemical modification, molecular programmability, and ability to selectively bind biological targets.^24^ DNA has been conjugated to various amphiphilic organic molecules including lipids,^25−30^ block copolymers,^31−38^ and dendrimers^39−41^ for applications ranging from membrane anchoring to nanostructure assembly.^41^ Conjugation of amphiphilic organic molecules to nucleic acid binding sequences, called aptamers, has resulted in useful materials for targeted delivery.^27,42,43^ Aptamers are selected through an iterative process called systematic evolution of ligands by exponential enrichment (SELEX) which utilizes either recombinant protein,^44,45^ live cells,^46−49^ or whole organisms^48,50^ as the selection target. Resulting aptamers not only bind target cells with high specificity, but they also enhance delivery of cargo into cells.^51^ In particular, aptamers have enhanced the uptake of various nanocarrier systems including LNPs,^52^ polymeric nanoparticles,^53^ and inorganic systems such as quantum dots and gold nanoparticles.^54^

**Scheme 1:**
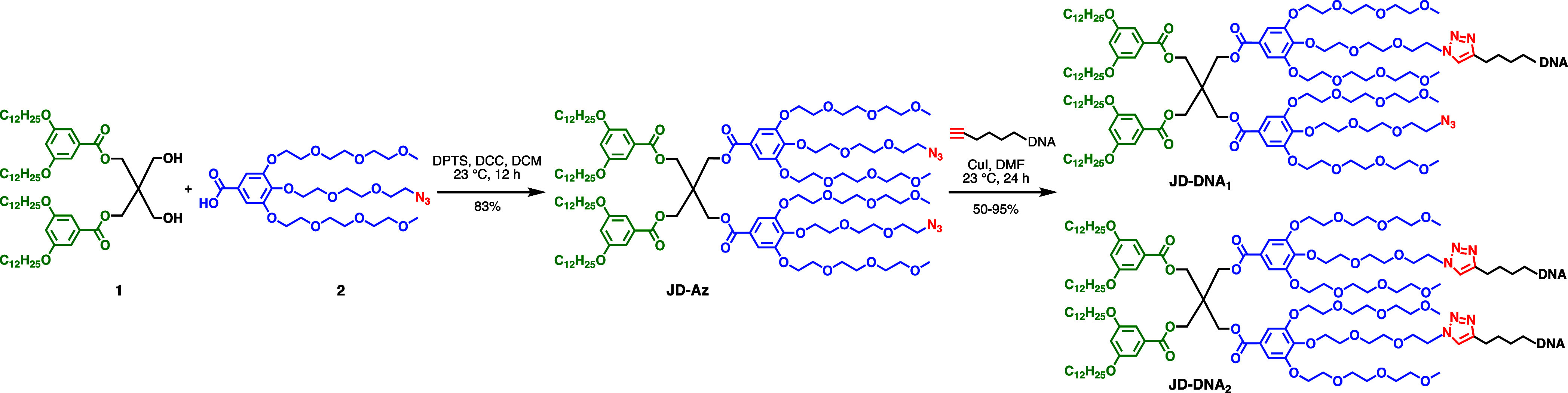
Synthesis of the Azido-Modified Janus Dendrimer (JD-Az) and DNA-Modified Janus Dendrimer (JD-DNA)

Here, we apply DNA-conjugation and aptamer targeting to Janus dendrimersomes as the next iteration of this technology. We report on the synthesis of azido-modified JDs that are conjugated to 16-nucleotide alkyne-modified DNA via copper-catalyzed azide alkyne cycloaddition (CuAAC), thus creating JD-DNA conjugates (Scheme 1). Although similar “click” reactions have been successful in DNA–DNA^55−58^ and DNA–polymer^59,60^ couplings in aqueous solution, fewer studies report success in organic solvent, which was utilized for our JD-DNA conjugation due to the hydrophobic nature of the azido-modified JD.^61^ JD-DNAs produced via “click” chemistry self-assemble into dendrimersomes displaying DNA anchors at the periphery, which are subsequently decorated with aptamers via DNA–DNA hybridization. In this way, we show that aptamers enhance the cellular uptake of dendrimersomes in vitro.

## Materials and Methods

### Synthesis of JD-Azide (JD-Az)

Compound **1** as the hydrophobic dendron^9^ and compound **2** as the hydrophilic dendron^13^ containing azide were synthesized and characterized according to the literature (Scheme 1). Reagents used for the synthesis of JD-Az from compound **1** and compound **2** were obtained from commercial sources and used without purification unless otherwise stated. CH_2_Cl_2_ (DCM) was dried over calcium hydride and freshly distilled before use. To this distilled DCM (10 mL), compound **1** (310 mg, 0.29 mmol), compound **2** (370 mg, 0.60 mmol), and 4-(dimethylamino)pyridinium 4-toluenesulfonate (DPTS, 176 mg, 0.60 mmol) were added, followed by *N,N′*-dicyclohexylcarbodiimide (DCC, 309 mg, 1.50 mmol). The mixture was allowed to stir at 23 °C for 12 h. Evolution of the reaction was monitored by thin-layer chromatography (TLC) using silica gel 60 F_254_-precoated plates (E. Merck), and compounds were visualized by UV light with a wavelength of 254 nm. The precipitate was filtered with Celite, and the filtrate was concentrated to dryness. The crude product was further purified by flash column chromatography using flash silica gel from Silicycle (60 Å, 40–63 μm) with a mobile phase of DCM/methanol = 10/1 (v/v) to yield compound **JD-Az** as a colorless oily liquid (550 mg, 83%). The chemical structure of JD-Az was confirmed by ^1^H and ^13^C NMR spectroscopy and MALDI-TOF (Figures S1–S3).

### Conjugation of JD-Azide to DNA-Alkyne

JD-Az was conjugated to alkyne-modified DNA (DNA-Alk: 5′ hexynyl-TTT TTT TAA GTG TAG T 3′) (Integrated DNA Technologies) using copper-catalyzed azide alkyne cycloaddition (CuAAC) or “click” chemistry, forming products **JD-DNA**_**1**_ and **JD-DNA**_**2**_ (Scheme 1). JD-Az (100 μM) and DNA-Alk (10 μM) were dissolved in either 100 or 1000 μL of DMF, depending on the reaction scale, and then mixed with a 1 mM iodo(triethyl phosphite) copper(I) (CuI) catalyst (Sigma-Aldrich).^31^ The “click” reaction was incubated for 24 h at room temperature. DMF was then removed using a speed vacuum concentrator. Dried JD-DNAs were resuspended in 0.1 M triethylamine acetate (TEAA) (Glen Research) so that JD concentration was maintained at 0.4 mM for subsequent purification. Samples in TEAA were heated at 65 °C for 5 min, immediately vortexed, and then cooled to room temperature.

### Purification of JD-DNA

JD-DNA products were analyzed and purified using reverse-phase high-performance liquid chromatography (HPLC) on the 1260 Infinity II LC System (Agilent). Samples were prepared in 90% 0.1 M TEAA with 10% acetonitrile (ACN) and heated to 65 °C before injection. Characterization and purification were carried out on an Xbridge Protein BEH C4 column (300 Å, 4.6 × 150 mm) (Waters) using a linear gradient of 10–100% ACN in 0.1 M TEAA over 20 min at 65 °C. Absorbance was monitored at 260 nm (Figure S4). Purified JD-DNA products (JD-DNA_1_ and JD-DNA_2_) were lyophilized and stored at −20 °C. DNA concentration was measured using a NanoDrop One Microvolume UV–Vis Spectrophotometer (Thermo Scientific). Masses of purified JD-DNA_1_ and JD-DNA_2_ were confirmed by liquid chromatography mass spectrometry (Figures S5–S8).

### Agarose Gel Electrophoresis

Gel electrophoresis was used to quantify JD-DNA conjugation yield as well as aptamer hybridization efficiency. Gels were prepared with 4% SeaKem LE agarose (Lonza) dissolved in 1× Tris-Borate-EDTA (TBE) buffer. SYBR Gold Nucleic Acid Gel Stain (1×) (Thermo Fisher Scientific) was added to the gel prior to polymerization. JD-DNA samples containing 2 μg of DNA in 0.1 M TEAA were prepared with 6× Orange Gel Loading Dye (New England Biolabs, Inc.) and loaded into the gel. The O’RangeRuler 10 bp DNA ladder (Thermo Fisher Scientific) was used to approximate DNA size. Gels were electrophoresed at 180 V and then imaged using the NuGenius gel documentation workstation (Syngene). Densitometry analysis was performed using ImageJ software (National Institutes of Health).

### Assembly of JD-DNA Dendrimersomes

Dendrimersomes were prepared via thin film hydration. Purified JD-DNA conjugates stored in either ethanol or 0.1 M TEAA were mixed with a dialkylcarbocyanine fluorophore, dried in a speed vacuum concentrator, and resuspended in 25 mM sodium phosphate buffer with 50 mM potassium chloride (pH 7.4) at a final DNA concentration of 20 μM. Samples were heated at 65 °C for 5 min, immediately vortexed, and cooled at room temperature. Dialkylcarbocyanine fluorophores, either DiO or DiI (Biotium), were prepared at 1 mM in DMF before mixing with the JD-DNA. Fluorophore concentration in dendrimersomes was maintained at 50 μM. DiO-labeled dendrimersomes were used in flow cytometry experiments, whereas DiI-labeled dendrimersomes were used for fluorescence microscopy.

### Hybridization of Aptamers to JD-DNA Dendrimersomes

JD-DNA dendrimersomes were hybridized to aptamers by mixing equal volumes of 20 μM dendrimersomes with a 20 μM aptamer. Aptamers were modified with a spacer (SpC6) and complementary DNA to the JD-DNA anchor sequence (Gene Link). Aptamers utilized include HG1–9 which binds the human transferrin receptor (HG1–9:5′ GGATAGGGATTCTG TTGGTCGGCTGGTTGGTATCC [SpC6] ACTACACTTAAAAAAA 3′) and a negative control aptamer (ctrl) for cell uptake experiments (ctrl: 5′ AGAGCAGCGTGGAGGATAG TTGGGGTTTGGCAAGTATTG [SpC6] ACTACACTTAAAA AAA 3′).^62^ The dendrimersome-aptamer mixture was heated to 65 °C for 5 min, cooled at room temperature for 5 min, and then cooled on ice for 5 min before use in in vitro experiments.

### Dynamic Light Scattering (DLS)

DLS was used to measure dendrimersome size distribution. Dendrimersomes were prepared as described above but without the dialkylcarbocyanine fluorophore. Samples were analyzed in disposable microcuvettes (Malvern Panalytical) on a Zetasizer Nano ZS instrument (Malvern Panalytical) at 25 °C.

### Cryogenic Electron Microscopy (cryo-EM)

JD-DNA_1_ and JD-DNA_2_ dendrimersomes, respectively, were prepared via thin film hydration as described above. For cryo-EM imaging, 3 μL of each sample was applied onto CFlat holey carbon grids and plunge frozen in liquid ethane using a Vitrobot Mark IV (Thermo Fisher Scientific). Images were taken on a Titan Krios G3i equipped with a K3 Bioquantum.

### HEK293T Cell Maintenance

Human embryonic kidney 293 T (HEK293T) cells were maintained in Dulbecco’s modified Eagle's medium (DMEM) containing high glucose and sodium pyruvate (Gibco) supplemented with 10% fetal bovine serum (FBS) (Gibco) and 100 U/mL penicillin–streptomycin with 0.292 mg/mL l-glutamine (Gibco). Cells were grown at 37 °C with 5% CO_2_ and 99% humidity. At ∼80% confluency, cells were washed with Dulbecco’s phosphate-buffered saline (DBPS) lacking calcium and magnesium, trypsinized using 0.25% trypsin-EDTA (Gibco), and either transferred to a new flask for growth or plated for subsequent flow cytometry or fluorescence microscopy experiments.

### Fluorescence Microscopy

Fluorescence microscopy was used to visualize aptamer-modified dendrimersome uptake. Prior to seeding cells for microscopy, 22 mm glass coverslips (Fisher Scientific) were coated with a 0.01% poly-l-lysine (PLL) solution (MilliporeSigma) and incubated in the solution for 5 min. Coverslips were washed twice in distilled water. Once excess water was removed, PLL-coated coverslips were placed into a sterile 6-well plate and air-dried for 1 h. HEK293T cells were then trypsinized as described above, counted, and seeded at 600,000 cells per well. Cells were incubated on coverslips for 2 days before beginning dendrimersome uptake experiments.

To begin uptake experiments, media from cells was removed, and cells were washed twice with DPBS. Binding buffer containing 50 mM MgCl_2_ (Sigma-Aldrich), 1 mg/mL purified DNA from salmon testes (MilliporeSigma), 45 mg/mL glucose (Sigma-Aldrich), and 10 mg/mL bovine serum albumin (Sigma-Aldrich) in DMEM was warmed to 37 °C, and then 1 mL was added to each well of the 6-well plate, followed by 200 nM of DiI-labeled dendrimersomes. Cells were incubated with dendrimersomes for 30 min at 37 °C. Afterward, binding buffer was removed, and cells were washed twice with DPBS. Cells were fixed with 1 mL of 3.7% formaldehyde (Fisher Scientific), incubated for 15 min, and washed twice with DPBS. Cell nuclei were stained by incubating fixed cells for 5 min in 1 μg/mL 4′,6-diamidino-2-phenylindole (DAPI) (Thermo Scientific) prepared in DPBS. Cells were then washed twice with DPBS. Coverslips were removed and mounted on microscope slides using Fluoromount-G mounting medium (Invitrogen).

Fluorescence microscopy was performed on fixed cells using an Eclipse 80i upright microscope (Nikon) equipped with a D-FL epi-fluorescence attachment (Nikon), a DS-Qi2 camera (Nikon), and the SOLA Light Engine light source (Lumencor). Images were collected using a 20×/0.75 numerical aperture (NA) objective and acquired using NIS-Elements AR software (Nikon). Standard filter sets were used for fluorescence imaging: (1) 375/28 nm excitation and 460/50 nm emission for DAPI and (2) 540/25 nm excitation and 605/55 nm emission for DiI. Imaging settings (laser power and exposure time) were kept constant across all images collected with the same filter set. ImageJ software (National Institutes of Health) was used to make composite figures and apply scale bars.

### Flow Cytometry

Flow cytometry was used to measure aptamer-modified dendrimersome uptake. HEK293T cells were seeded at 250,000 cells per well in a 24-well plate 24 h before the uptake experiment. Media was then removed, and cells were washed twice with DPBS. Binding buffer, prepared as described above, was warmed to 37 °C and added to cells, followed by 200 nM of DiO-labeled dendrimersomes. Cells were incubated with dendrimersomes for 30 min at 37 °C, washed twice with DPBS, trypsinized, and resuspended in flow cytometry buffer consisting of Hank’s balanced salt solution (HBSS) (Gibco) with calcium and magnesium supplemented with 1% BSA and 0.1% sodium azide. Following centrifugation at 300 g for 3 min, cells were resuspended in flow cytometry buffer containing 0.5 μg/mL 7-amino-actinomycin D (7AAD) (Invitrogen) to assess cell viability. Flow cytometry was performed on a FACSCantoII (BD Biosciences). Samples were excited at 488 nm, and detection was monitored at 695/40 nm for 7-AAD and 525/50 nm for DiO.

## Results and Discussion

### Synthesis and Characterization of JD-DNA Conjugates

The azido-modified JD (JD-Az) was synthesized in a one-step reaction using previously reported minidendron building blocks.^9,13^ The azido-functionalized hydrophilic minidendron^13^ was conjugated to the hydrophobic ethylene glycol-dodecane dendron^9^ via *N,N*′-dicyclohexylcarbodiimide (DCC) and 4-(dimethylamino)pyridinium 4-toluenesulfonate (DPTS) in dichloromethane (DCM) at room temperature (Scheme 1).^63^ The resulting amphiphilic JD-Az was produced in 83% yield and contained two azido functional groups, one per hydrophilic minidendron. Using CuAAC, JD-Az was modified with short 16-nucleotide alkyne-modified DNA sequences (DNA-Alk), producing JD-DNA conjugates bearing either one (JD-DNA_1_) or two DNA (JD-DNA_2_) sequences per JD (Scheme 1).

**Figure 1:**
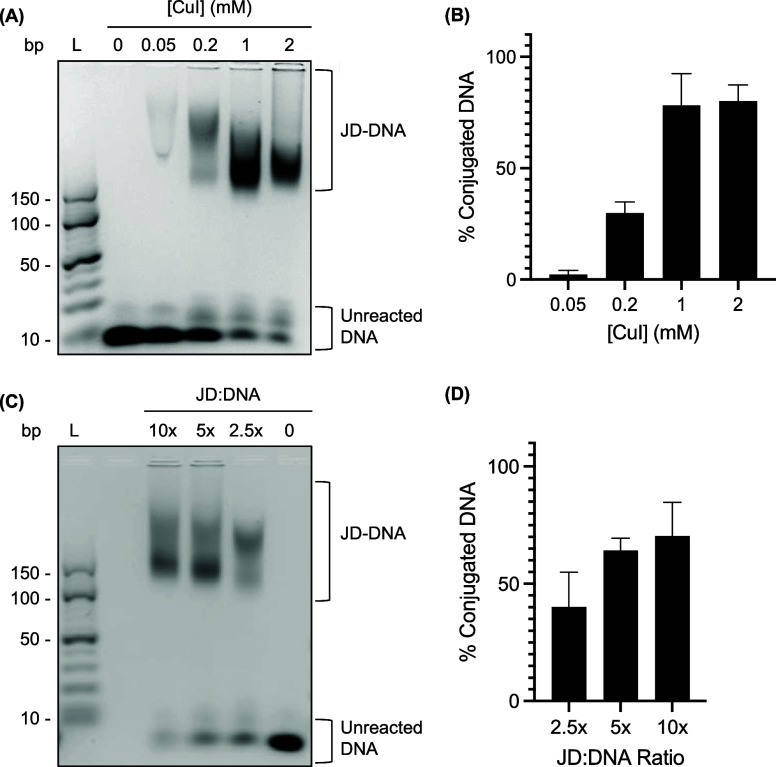
Optimization of the JD-DNA click reaction. (A) Agarose gel demonstrating successful synthesis of JD-DNA with increasing amounts of the iodo(triethyl phosphite) copper(I) (CuI) catalyst. Ladder (L) depicts O’Range Ruler 10 base pair (bp) DNA ladder. (B) Quantification of the percentage of JD-DNA conjugate relative to unreacted DNA observed by gel electrophoresis (*n* = 3). Error bars represent standard error of the mean (SEM). (C) Agarose gel demonstrating JD-DNA yield in the presence of 1 mM CuI with 10×, 5×, or 2.5× molar excess of JD-Az in comparison to DNA-Alk. (D) Quantification of the percentage of JD-DNA conjugate relative to unreacted DNA observed by gel electrophoresis (*n* = 2). Error bars represent standard error of the mean (SEM).

The hydrophobic nature of the JD-Az required consideration of the solvent selected for CuAAC. In accordance with Wilks et al., CuAAC for JD-Az and DNA-Alk conjugation was performed in dimethylformamide (DMF) in the presence of an active copper catalyst, iodo(triethyl phosphite) copper(I) (CuI) (Scheme 1).^31^ The CuI catalyst concentration was varied from 0 to 2 mM to optimize JD-DNA conjugation efficiency (Figure 1A). Based on gel electrophoresis, 1 mM CuI maximized JD-DNA production with yields ranging from 50 to 95% while utilizing the least amount of catalyst (Figure 1B). A similar organic-soluble copper catalyst, bromotris(triphenylphosphine) copper(I), was ineffective at producing JD-DNA conjugates (data not shown).^31,64^ Copper-free reaction conditions using JD-Az and dibenzocyclooctyl-modified (DBCO) DNA in DMF were also unsuccessful (data not shown).^65^ Therefore, using 1 mM CuI, the ratio of JD-Az to DNA-Alk was varied from 10:1 to 5:1 to 2:1, with a 10-fold excess of JD-Az to DNA-Alk providing optimal reaction conditions by minimizing unreacted DNA-Alk (Figure 1C,D). After 24 h incubation at room temperature, JD-DNA_1_ and JD-DNA_2_ were purified from the reaction mixture using reverse-phase high performance liquid chromatography (HPLC) (Figure S4). Molecular weights of both products were verified via liquid chromatography–mass spectrometry with JD-DNA_1_ at 7348.294 Da and JD-DNA_2_ at 12,413.134 Da (Figures S5–S8).

### Janus Dendrimersome Assembly and Size Distribution

JD-DNA was used to assemble dendrimersomes via thin film hydration. Purified JD-DNA solutions were dried in a speed vacuum concentrator, resuspended in 25 mM sodium phosphate buffer with 50 mM potassium chloride (pH 7.4), and heated at 65 °C. The resulting dendrimersomes were analyzed by DLS to determine their diameter (*d*, nm) and polydispersity index (PDI). Dendrimersomes made from JD-DNA_1_ were 73.3 nm (PDI 0.384) and those made from JD-DNA_2_ were 115.2 nm (PDI 0.297) (Table 1 and Figure S9). Cryo-EM was also performed to observe vesicle formation. Preliminary results suggest that JD-DNA_1_ and JD-DNA_2_, respectively, self-assemble into vesicles or dendrimersomes (Figure S10). These observations are in accordance with previous studies that document vesicle formation from various JD libraries using cryo-EM, including those containing the hydrophilic and hydrophobic components of JD-Az.^9,13,15,66,67^ Taken together, DLS and cryo-EM support that this facile method for preparing dendrimersomes results in vesicle formation, and these vesicles are similar in size to other nanocarrier delivery systems.^68^

**Table 1:** Size Distributions of JD-DNA Dendrimersomes via DLS

	*Z*-average size (d, nm)	polydispersity index (PDI)
JD-DNA_1_	73.3	0.384
JD-DNA_2_	115.2	0.297

### Aptamer Hybridization to Janus Dendrimersomes

The ultimate goal of this work is to demonstrate that JD-DNA dendrimersomes can be delivered to cells in a targeted manner since precise delivery of nanocarriers to specific cells is necessary to reduce off-target effects.^1^ The human transferrin receptor is a particularly useful target because it is overexpressed on many cancer cells and is also a target for traversing cargo across the blood–brain barrier.^69−71^ Furthermore, transferrin receptor aptamers conjugated to PEGylated lipids have been successful at enhancing the uptake of lipid-based nanoparticles.^52^ Here, we have chosen a DNA aptamer, HG1–9, which has been reported to bind the human transferrin receptor (hTfR) in malignant cells and to compete with human transferrin (hTf) for binding, indicating that the aptamer and hTf bind the receptor at a similar location.^62,72^ Moreover, the binding affinity of HG1–9 for hTfR in HeLa and Jurkat cells (11.0 ± 2.9 and 19.8 ± 4.9 nM, respectively) is on the same order of magnitude as reported literature values for apo-hTf, further suggesting its utility as an effective targeting ligand.^62,73^

**Figure 2:**
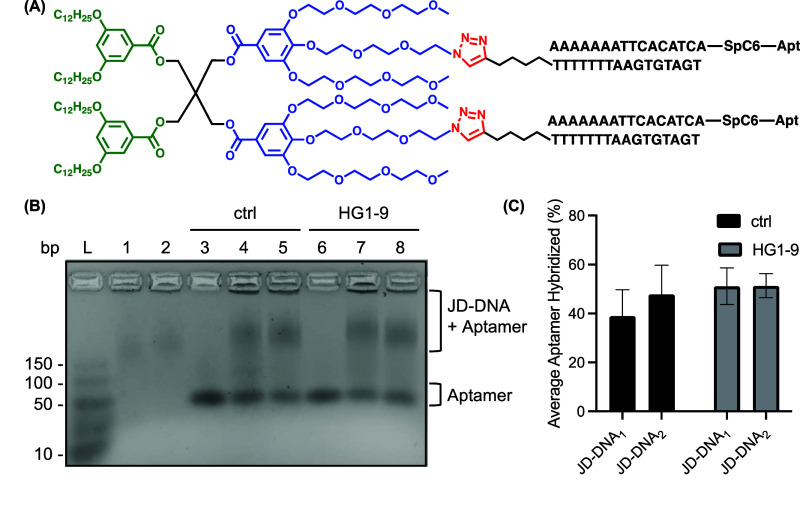
Aptamer hybridization to JD-DNA dendrimersomes. (A) Representation of JD-DNA_2_ hybridized to complementary DNA covalently modified with a six-carbon spacer (SpC6) and an aptamer (Apt). (B) Agarose gel electrophoresis depicts aptamer hybridization to JD-DNA dendrimersomes where lanes represent (1) JD-DNA_2_, (2) JD-DNA_1_, (3) ctrl aptamer, (4) JD-DNA_2_ + ctrl, (5) JD-DNA_1_ + ctrl, (6) HG1–9 aptamer, (7) JD-DNA_2_ + HG1–9, and (8) JD-DNA_1_ + HG1–9. Ladder (L) depicts O’Range Ruler 10 bp DNA ladder. (C) Quantification of average aptamer hybridization observed via gel electrophoresis (*n* = 3). Error bars represent SEM.

To hybridize aptamers to JD-DNA dendrimersomes, HG1–9 was modified at the 3′ end with a six-carbon spacer (SpC6) and a 16-nucleotide sequence that was complementary to the DNA anchor displayed on the dendrimersome surface. A negative control aptamer (ctrl), previously reported to not bind malignant cells, was similarly modified with SpC6 and the complementary DNA.^62^ Dendrimersomes assembled from either JD-DNA_1_ or JD-DNA_2_ were incubated with an equimolar concentration of aptamer at 65 °C for 5 min and then slowly cooled to promote aptamer hybridization (Figure 2A). Hybridization of HG1–9 and ctrl aptamers to both JD-DNA_1_ and JD-DNA_2_ dendrimersomes was confirmed using gel electrophoresis (Figure 2B). Average aptamer hybridization efficiency across all samples was 47.3 ± 5.8%, as determined by gel electrophoresis, likely because DNA anchor sequences may not only be displayed on the exterior of the dendrimersome but may also be present in the interior (Figure 2C).

### Analysis of Dendrimersome Uptake

**Figure 3:**
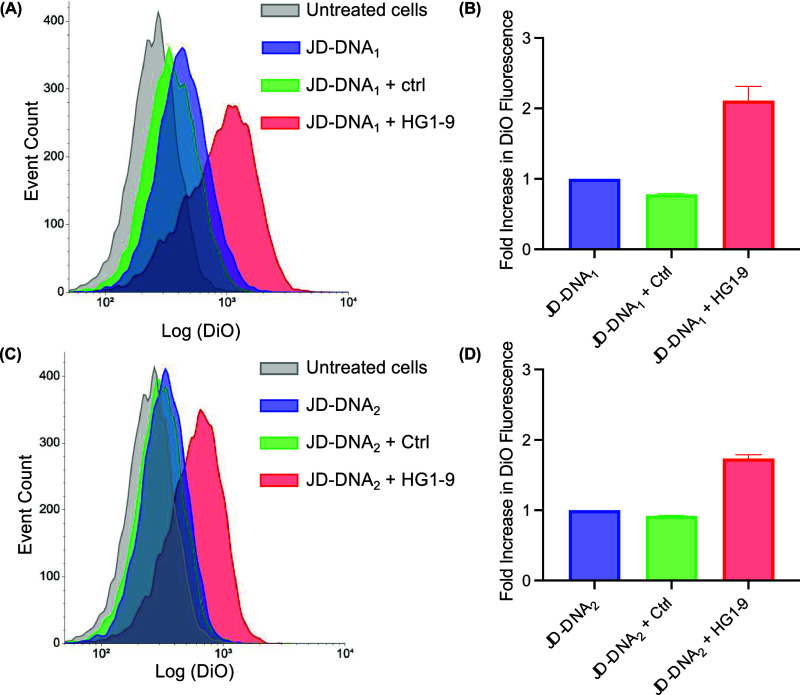
Uptake of JD-DNA dendrimersomes in HEK293T cells, as measured by flow cytometry. DiO-labeled dendrimersomes were incubated with HEK293Ts for 30 min at 37 °C in binding buffer. Cells were then washed and stained with 7-AAD. Representative histograms displaying event count versus increase in DiO fluorescence for cells that were either untreated (gray) or treated with (A) JD-DNA_1_ or (C) JD-DNA_2_ dendrimersomes bearing no aptamer (blue), ctrl aptamer (green), or HG1–9 aptamer (red). Quantification of fold increase in mean DiO fluorescence for (B) aptamer-hybridized JD-DNA_1_ dendrimersomes and (D) aptamer-hybridized JD-DNA_2_ dendrimersomes as compared to cells treated with either JD-DNA_1_ or JD-DNA_2_ without an aptamer, respectively (*n* = 3). Error bars represent SEM.

Cellular uptake of aptamer-hybridized dendrimersomes was assessed using both flow cytometry and fluorescence microscopy. Dendrimersomes prepared for these studies were labeled with dialkylcarbocyanine fluorophores during thin film hydration, with DiO being used for flow cytometry and DiI for fluorescence microscopy. Human embryonic kidney 293 T cells (HEK293T) were treated with JD-DNA_1_ or JD-DNA_2_ dendrimersomes bearing no aptamer, ctrl aptamers, or HG1–9 aptamers for 30 min at 37 °C in binding buffer containing 50 mM MgCl_2_, 1 mg/mL purified DNA from salmon testes, 45 mg/mL glucose, and 10 mg/mL bovine serum albumin. These binding conditions mimic the environment in which the HG1–9 aptamer was selected, with salmon testes DNA serving as a blocking agent for nonspecific DNA interactions with the cell surface.^62,72,74^ Cells were then trypsinized, stained with 7-amino-actinomycin D (7AAD) to assess cell viability, and analyzed by flow cytometry to determine dendrimersome uptake via an increase in DiO fluorescence (Figure 3). Compared to cells treated with dendrimersomes bearing no aptamer, dendrimersomes with HG1–9 aptamers show enhanced cellular uptake, with a 2.11 ± 0.35 or a 1.73 ± 0.10 fold-increase in DiO fluorescence for JD-DNA_1_ and JD-DNA_2_ dendrimersomes, respectively (Figure 3B,D). Variation in uptake for JD-DNA_1_ and JD-DNA_2_ dendrimersomes may be due to differences in aptamer display on each dendrimersome sample or due to differences in dendrimersome diameter (Table 1).^75^ Aptamer surface concentration, distance between adjacent aptamers, and geometric arrangement of aptamers on the dendrimersome surface are factors that may affect aptamer binding to the transferrin receptor, resulting in differences between JD-DNA_1_ and JD-DNA_2_ cell uptake.^76^ Regardless, these quantitative results suggest that HG1–9 is needed for the uptake of JD-DNA dendrimersomes.

**Figure 4:**
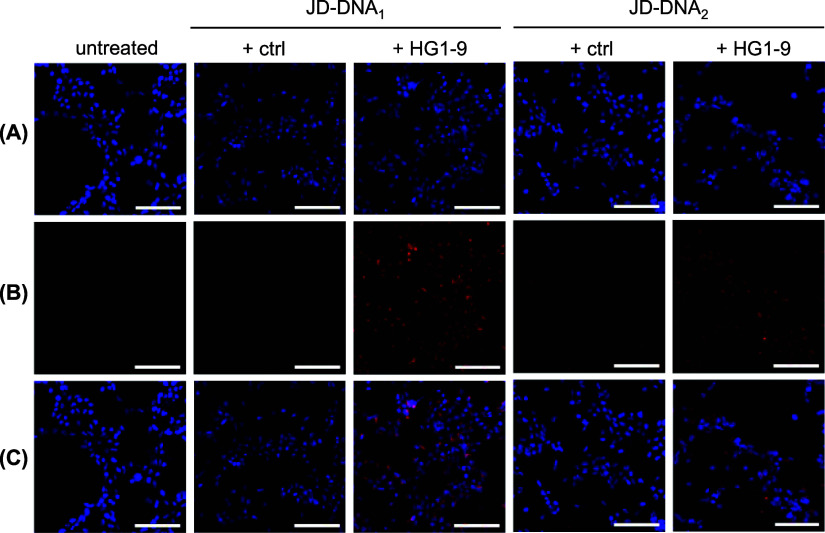
Visualization of JD-DNA dendrimersome uptake in HEK293T cells using fluorescence microscopy. DiI-labeled dendrimersomes were incubated with HEK293Ts for 30 min at 37 °C in binding buffer. Cells were then washed, fixed in formaldehyde, and stained with DAPI. Representative images show cell nuclei in blue (A), dendrimersome uptake in red (B), and merged images (C). Scale bars represent 100 μm.

These results were corroborated via fluorescence microscopy. HEK293T cells were grown on glass coverslips before treatment with DiI-labeled JD-DNA dendrimersomes in binding buffer at 37 °C for 30 min. Cells were fixed in formaldehyde, stained with 4′,6-diamidino-2-phenylindole (DAPI), and mounted on microscope slides for imaging (Figure 4). DAPI was used to identify cell nuclei (Figure 4A), whereas DiI served as a fluorescent marker for dendrimersome uptake (Figure 4B). By visual inspection, JD-DNA_1_ and JD-DNA_2_ dendrimersomes hybridized to HG1–9 aptamers exhibited increased uptake as compared to untreated cells and those treated with either JD-DNA_1_ or JD-DNA_2_ dendrimersomes bearing ctrl aptamers, further demonstrating that the HG1–9 aptamer enhances dendrimersome uptake in vitro.

## Conclusions

We have synthesized an azido-modified JD that can be utilized in CuAAC reactions for conjugation to DNA. These JD-DNA conjugates assemble into vesicles, or dendrimersomes, with diameters similar to other nanocarrier systems, as confirmed by DLS and preliminary cryo-EM analysis.^68^ Importantly, the DNA sequence conjugated to the JD can serve as a hybridization scaffold for the addition of aptamers. We have used HG1–9, the hTfR-binding DNA aptamer, as proof-of-concept to show that aptamers can increase dendrimersome uptake in target cells. The nanocarrier system outlined in this report is an attractive platform for future drug delivery applications. The simplicity of the JD-DNA “click” reaction provides the opportunity for conjugation of nucleic acids of various sequences and lengths. Hybridization of complementary DNA strands to JD-DNA dendrimersomes via heating and cooling is equally facile and may allow for the hybridization of other target ligands or may also find utility in delivery of oligonucleotide therapeutics, either via hybridization or direct conjugation to the JD. By exploring the structural space of the JD-Az in conjunction with various DNA modifications, new delivery applications may be discovered for these Janus dendrimersomes.

## Supporting Information Available

NMR and MALDI-TOF methods and analysis of JD-Az, HPLC chromatogram of JD-DNA purification, LC/MS methods and analysis of JD-DNAs, dendrimersome size distributions via DLS, and cryo-EM images (PDF)
