# Supplementary material for: Aptamer-Targeted Dendrimersomes Assembled from Azido-Modified Janus Dendrimers “Clicked” to DNA
Source: Biomacromolecules. 2024 Feb 23;25(3):1541–9. doi: 10.1021/acs.biomac.3c01108 (PMC10934268; doi:10.1021/acs.biomac.3c01108)
Supplement: Supplementary file 1 — bm3c01108_si_001.pdf [file bm3c01108_si_001.pdf]

Supporting Information for

Aptamer-Targeted Dendrimersomes Assembled from Azido-Modified Janus Dendrimers "Clicked" to DNA

Paige Bristow,[1] Kyle Schantz,[1] Zoe Moosbrugger,[1] Kailey Martin,[1] Haley Liebenberg,[1] Stefan Steimle,[2] Qi Xiao,[3] Virgil Percec,[3] and Samantha E. Wilner[1]*

[1] Department of Chemistry, Ursinus College, Collegeville, Pennsylvania 19426, United States

[2] Department of Biochemistry and Biophysics, Perelman School of Medicine, University of Pennsylvania, Philadelphia, PA 19014

[3] Roy & Diana Vagelos Laboratories, Department of Chemistry, University of Pennsylvania, Philadelphia, Pennsylvania 19104, United States

*Email: swilner@ursinus.edu

Table of Contents

1. ^{1}H and ^{13}C NMR Spectroscopy Analysis of JD-Az 3
2. Matrix Assisted Laser Desorption Ionization-Time of Flight (MALDI-TOF) Mass Spectrometry Analysis of JD-Az 4
3. Reverse-Phase High Performance Liquid Chromatography (HPLC) Purification of JD-DNA Click Reaction 5
4. Liquid Chromatography Mass Spectrometry (LC/MS) Analysis of JD-DNA 5
5. Dendrimersome Size Distribution 7
6. Cryogenic Electron Microscopy (cryo-EM) Analysis of Dendrimersomes.................. 8

1. ^{1}H and ^{13}C NMR Spectroscopy Analysis of JD-Az

^{1}H and ^{13}C NMR spectra for **JD-Az** were recorded at 500 MHz and 126 MHz respectively, on a Bruker DRX (500 MHz) NMR spectrometer at 23 °C. Chemical shifts (δ) are reported in ppm and coupling constants (J) are reported in Hertz (Hz). The resonance multiplicities in the ^{1}H NMR spectra are described as "s" (singlet), "d" (doublet), "t" (triplet), and "m" (multiplet). Residual protic solvent of $CDCl_3$ (^{1}H, δ 7.26 ppm; ^{13}C, δ 77.16 ppm, middle of the triplet), and tetramethylsilane (TMS, δ 0 ppm) were used as the internal reference in the ^{1}H and ^{13}C NMR spectra. NMR spectra were analyzed and exported by TopSpin 4.07 (Bruker). The absorptions are given in wavenumbers (cm^{-1}).

Successful synthesis of **JD-Az** was confirmed by ^{1}H and ^{13}C NMR spectroscopy according to the following results: ^{1}H NMR (500 MHz, $CDCl_3$) δ = 7.25 (s, 4H, $4\times Ar\mathit{H}$), 7.09 (d, 4H, J = 2.2 Hz, $4\times Ar\mathit{H}$), 6.62 (t, 2H, J = 2.2 Hz, $2\times Ar\mathit{H}$), 4.62 (s, 4H, $2\times COOC\mathit{H}_2$), 4.59 (s, 4H, $2\times COOC\mathit{H}_2$), 4.22–4.24 (t, 4H, J = 4.8 Hz, $2\times OC\mathit{H}_2CH_2OCH_2CH_2OCH_2CH_2N_3$), 4.17–4.18 (t, 8H, J = 4.8 Hz, $4\times OC\mathit{H}_2CH_2OCH_2CH_2OCH_2CH_2N_3$), 3.86–3.88 (t, 8H, J = 4.8 Hz, $4\times OCH_2C\mathit{H}_2OCH_2CH_2OCH_2CH_2OCH_3$), 3.80–3.82 (t, 4H, J = 4.8 Hz, $2\times OCH_2C\mathit{H}_2OCH_2CH_2OCH_2CH_2N_3$), 3.72–3.75 (m, 12H, $6\times C\mathit{H}_2$), 3.63–3.67 (m, 24H, $12\times C\mathit{H}_2$), 3.52–3.54 (m, 8H, $4\times OCH_2CH_2OCH_2CH_2OCH_2C\mathit{H}_2OCH_3$), 3.36–3.39 (m, 16H, $2\times OC\mathit{H}_2CH_2OCH_2CH_2OCH_2C\mathit{H}_2N_3$ and $4\times OCH_2CH_2OCH_2CH_2OCH_2CH_2OC\mathit{H}_3$), 1.74–1.79 (m, 8H, $4\times OCH_2C\mathit{H}_2CH_2(CH_2)_8CH_3$), 1.41–1.47 (m, 8H, $4\times OCH_2CH_2C\mathit{H}_2(CH_2)_8CH_3$), 1.26–1.34 (m, 64H, $4\times OCH_2CH_2CH_2(C\mathit{H}_2)_8CH_3$), 0.86–0.89 (m, 12H, $4\times OCH_2CH_2CH_2(CH_2)_8C\mathit{H}_3$). ^{13}C NMR (126 MHz, $CDCl_3$) δ = 165.9, 165.5, 160.3, 152.5, 143.0, 131.1, 124.2, 109.0, 107.8, 106.6, 72.6, 72.0, 70.9, 70.8, 70.8, 70.7, 70.6, 70.1, 69.7, 69.0, 68.4, 63.1, 63.0, 59.1, 50.8, 43.4, 32.0, 29.8, 29.7, 29.7, 29.5, 29.4, 29.3, 26.1, 22.8, 14.2.

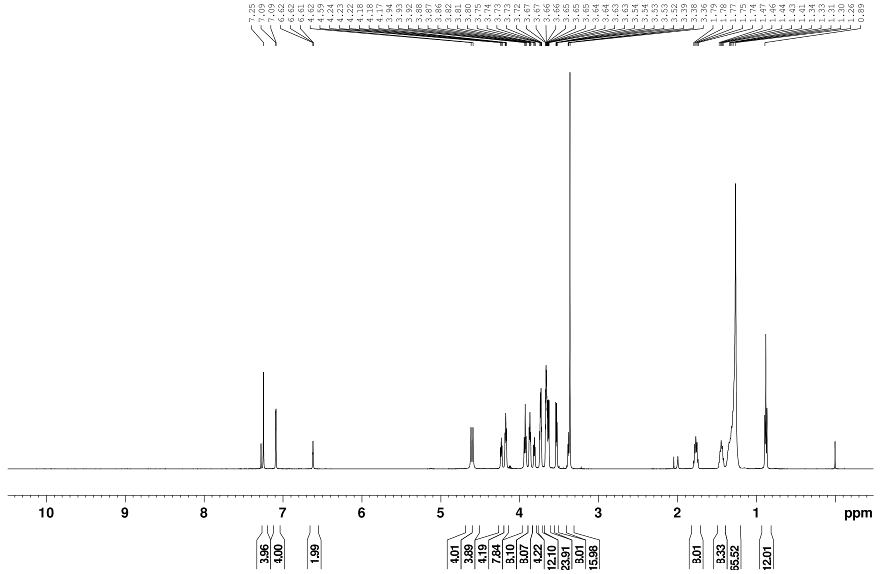

Figure S1. ^{1}H NMR spectrum of JD-Az ($CDCl_3$, 500 MHz).

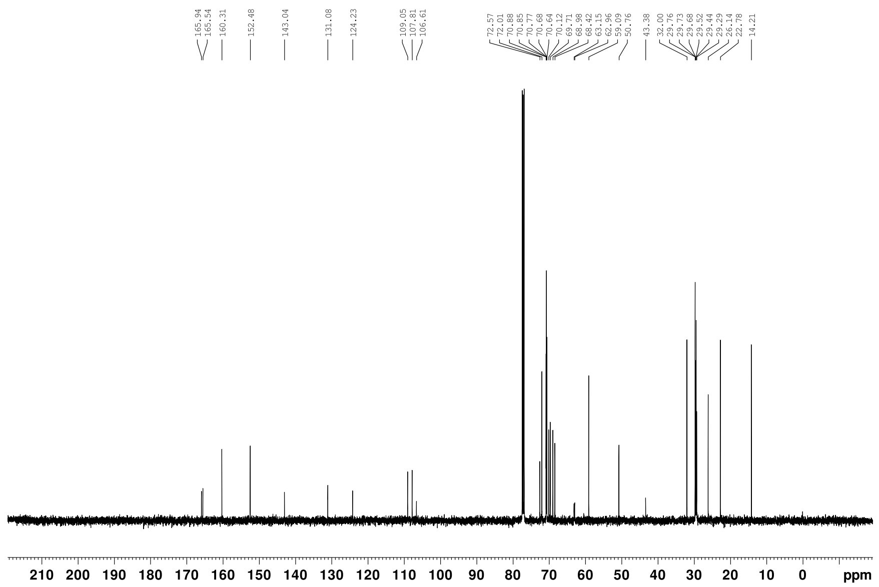

Figure S2. ^{13}C NMR spectrum of JD-Az ($CDCl_3$, 126 MHz).

2. Matrix Assisted Laser Desorption Ionization-Time of Flight (MALDI-TOF) Mass Spectrometry Analysis of JD-Az

MALDI-TOF mass spectrometry to confirm **JD-Az** mass was performed on a PerSeptive Biosystem-Voyager-DE MALDI-TOF mass spectrometer equipped with a nitrogen laser (337 nm) and operating in linear positive-ion mode. The analytical sample was obtained by mixing JD-Az (20–25 mg/mL) in dimethylformamide (DMF) with a THF solution of the matrix (2,5-dihydroxybenzoic acid, 10 mg/mL) in a 1/5 (v/v) ratio. The prepared solution of the sample and the matrix (0.5 µL) was loaded on the MALDI plate and allowed to dry at 23 ºC before the plate was inserted into the vacuum chamber of the MALDI instrument. The low mass gate was set to 500 Da, the extraction delay time was 200 ns, and the accelerating voltage was 20000 V. Mass spectra were acquired in a *m/z* range 600-4000 Da with 100 laser shots per spectrum. Expected *m/z* of $[M+Na]^+$ calculated for **JD-Az**: 2307.9 Da; observed $[M+Na]^+$: 2307.2 Da.

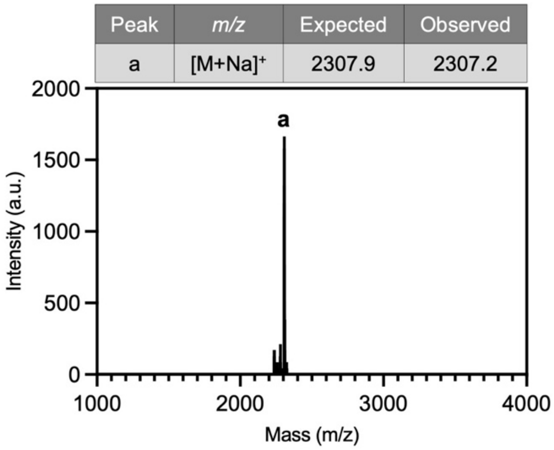

Figure S3. MALDI-TOF mass spectrum of JD-Az. Expected *m/z* of $[M+Na]^+$ calculated for JD-Az: 2307.9 Da. Observed $[M+Na]^+$: 2307.2 Da.

3. Reverse-Phase High Performance Liquid Chromatography (HPLC) Purification of JD-DNA Click Reaction

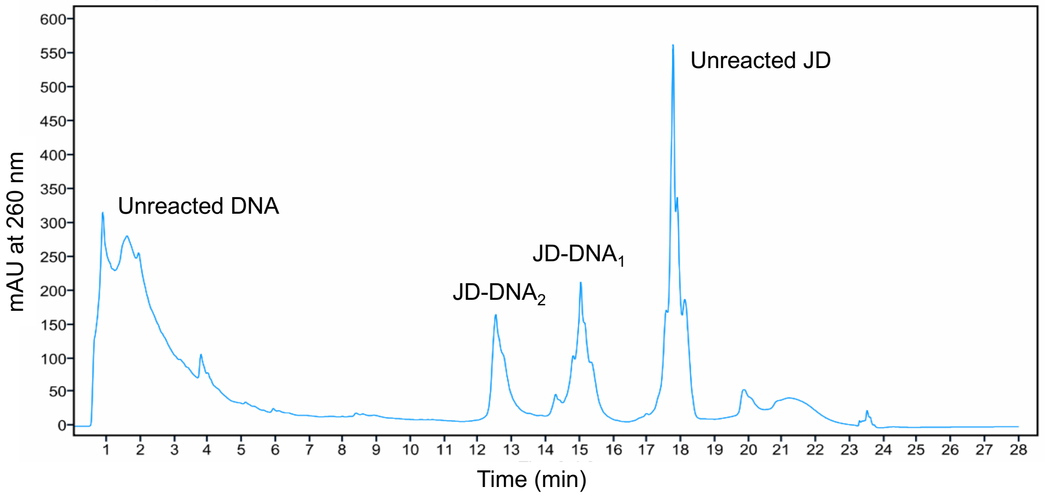

Figure S4: Representative reversed-phase HPLC chromatogram of the JD-DNA click reaction monitored at 260 nm.

4. Liquid Chromatography Mass Spectrometry (LC/MS) Analysis of JD-DNA

JD-DNA$_1$ and **JD-DNA$_2$** masses were confirmed by electrospray ionization LC/MS. High Resolution/Accurate Mass (HRMS) LC/MS analysis was performed by Novatia, LLC using their Oligo high-throughput characterization system (HTCS). Data was processed using their ProMass automated biomolecule deconvolution software.

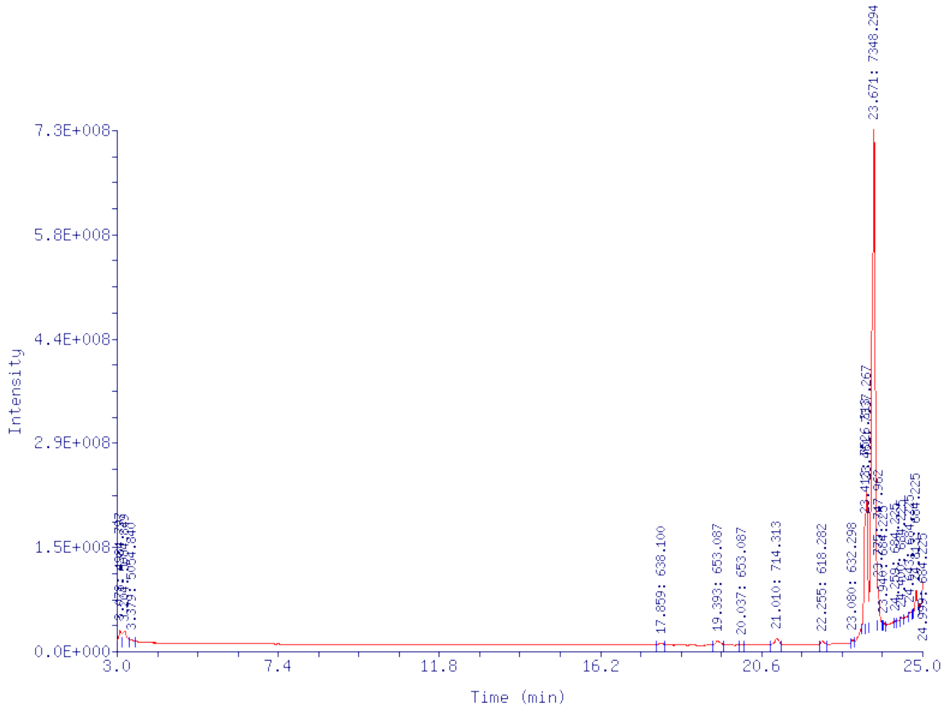

Figure S5. LC/MS chromatogram for JD-DNA$_1$.

Peak	Expected Mass (Da)	Observed Mass (Da)
a	7348.290	7348.294

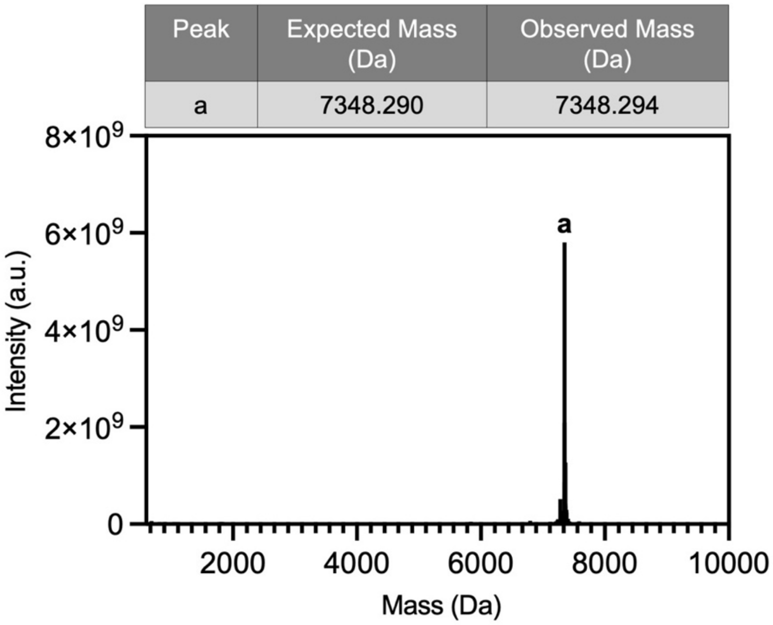

Figure S6. Deconvoluted mass spectrum of major retention peak (23.671 minutes) for JD-DNA$_1$ generated using ProMass software. Expected mass: 7348.290 Da. Observed mass: 7348.294 Da.

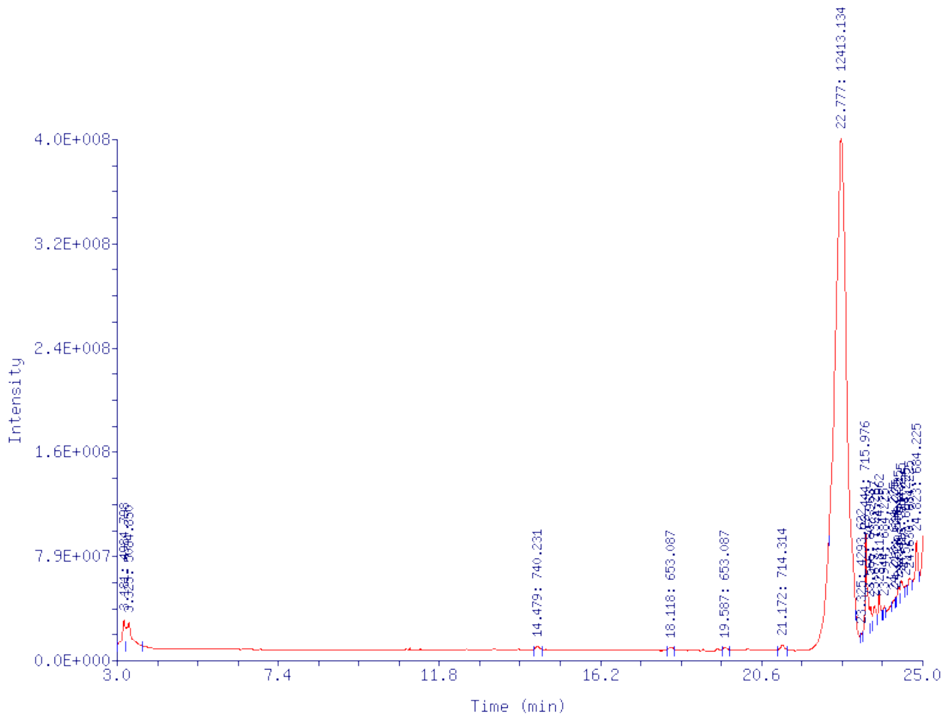

Figure S7. LC/MS chromatogram for JD-DNA$_2$.

Peak	Expected Mass (Da)	Observed Mass (Da)
a	12413.124	12413.134

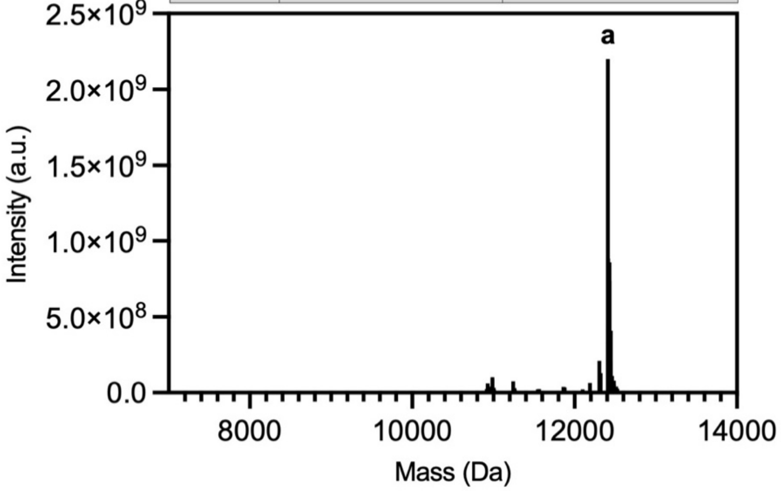

Figure S8. Deconvoluted mass spectrum of major retention peak (22.777 minutes) for JD-DNA_2 generated using ProMass software. Expected mass: 12413.124 Da. Observed mass: 12413.134 Da.

5. Dendrimersome Size Distribution

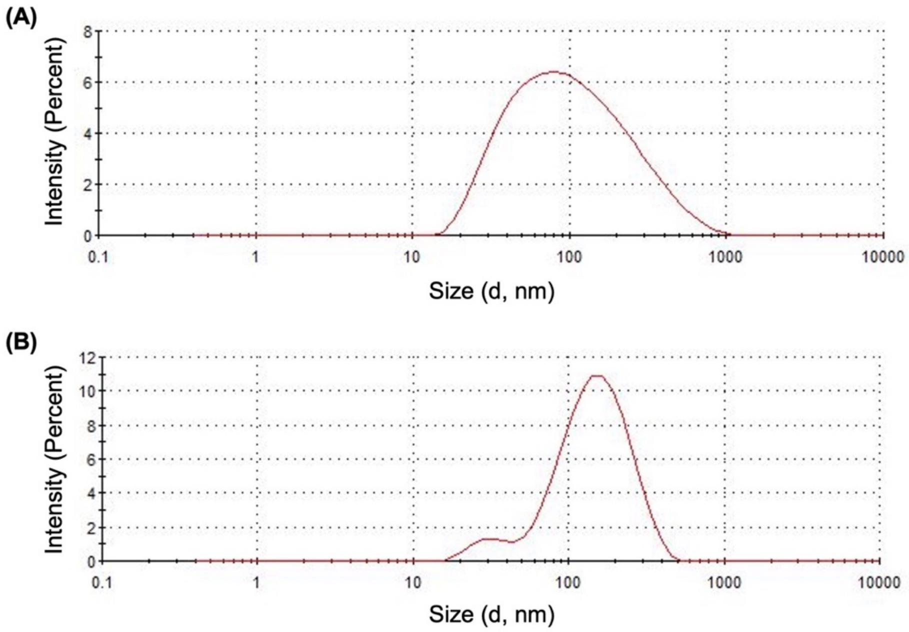

Figure S9. Representative DLS data of size distributions for (A) JD-DNA_1 dendrimersomes and (B) JD-DNA_2 dendrimersomes in 25 mM sodium phosphate buffer with 50 mM potassium chloride (pH 7.4).

6. Cryogenic Electron Microscopy (cryo-EM) Analysis of Dendrimersomes

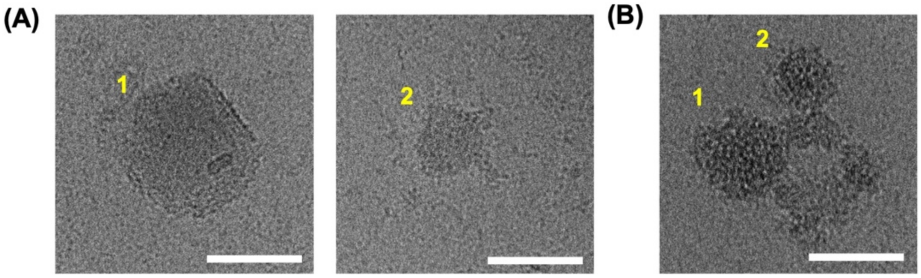

Figure S10. Representative cryo-EM images of dendrimersomes assembled from (A) JD-DNA_1 and (B) JD-DNA_2. Dendrimersomes are numbered in yellow. Scale bars represent 50 nm.
